# Supplementary material for: Is Bilirubin Associated with the Severity of Ischemic Stroke? A Dose Response Meta-Analysis
Source: J Clin Med. 2022 Jun 7;11(12):3262. doi: 10.3390/jcm11123262 (PMC9224549; doi:10.3390/jcm11123262)
Supplement: Supplementary file 1 [file jcm-11-03262-s001.zip › jcm-1682133-supplementary.pdf]

Supplement Table S1	**PRISMA checklist.**
Supplement Table S2	**Strategies for the database search**
Supplement Table S3	**Quality evaluation of included studies**
Supplement Table S4	**Characteristics of included studies**
Supplement Table S5	**Sensitivity analysis on the pooled ORs of ischemic stroke severity**
Supplement Table S6	**Effect estimates corresponding to each dose point**

Table S1. PRISMA checklist.

Section and Topic	Item #	Checklist item	Location where item is reported
**TITLE**			
Title	1	Identify the report as a systematic review.	Title
**ABSTRACT**			
Abstract	2	See the PRISMA 2020 for Abstracts checklist.	Supplement Table S1
**INTRODUCTION**			
Rationale	3	Describe the rationale for the review in the context of existing knowledge.	Line 17-29
Objectives	4	Provide an explicit statement of the objective(s) or question(s) the review addresses.	Line 29-33
**METHODS**			
Eligibility criteria	5	Specify the inclusion and exclusion criteria for the review and how studies were grouped for the syntheses.	Line 49-53
Information sources	6	Specify all databases, registers, websites, organisations, reference lists and other sources searched or consulted to identify studies. Specify the date when each source was last searched or consulted.	Line 53-55
Search strategy	7	Present the full search strategies for all databases, registers and websites, including any filters and limits used.	Supplementary Table S2
Selection process	8	Specify the methods used to decide whether a study met the inclusion criteria of the review, including how many reviewers screened each record and each report retrieved, whether they worked independently, and if applicable, details of automation tools used in the process.	Line 43-50
Data collection	9	Specify the methods used to collect data from reports, including how many reviewers collected data from each report,	Line 57-58

Section and Topic	Item #	Checklist item	Location where item is reported
process		whether they worked independently, any processes for obtaining or confirming data from study investigators, and if applicable, details of automation tools used in the process.	
Data items	10a	List and define all outcomes for which data were sought. Specify whether all results that were compatible with each outcome domain in each study were sought (e.g. for all measures, time points, analyses), and if not, the methods used to decide which results to collect.	Line 59-66
	10b	List and define all other variables for which data were sought (e.g. participant and intervention characteristics, funding sources). Describe any assumptions made about any missing or unclear information.	Line 67-70
Study risk of bias assessment	11	Specify the methods used to assess risk of bias in the included studies, including details of the tool(s) used, how many reviewers assessed each study and whether they worked independently, and if applicable, details of automation tools used in the process.	Line 64-66
Effect measures	12	Specify for each outcome the effect measure(s) (e.g. risk ratio, mean difference) used in the synthesis or presentation of results.	Line 90-92
Synthesis methods	13a	Describe the processes used to decide which studies were eligible for each synthesis (e.g. tabulating the study intervention characteristics and comparing against the planned groups for each synthesis (item #5)).	Line 93-95
	13b	Describe any methods required to prepare the data for presentation or synthesis, such as handling of missing summary statistics, or data conversions.	Line 96-100
	13c	Describe any methods used to tabulate or visually display results of individual studies and syntheses.	Line 103-105
	13d	Describe any methods used to synthesize results and provide a rationale for the choice(s). If meta-analysis was performed, describe the model(s), method(s) to identify the presence and extent of statistical heterogeneity, and software package(s) used.	Line 105-106

Section and Topic	Item #	Checklist item	Location where item is reported
	13e	Describe any methods used to explore possible causes of heterogeneity among study results (e.g. subgroup analysis, meta-regression).	Line 109-110
	13f	Describe any sensitivity analyses conducted to assess robustness of the synthesized results.	Line 109-110
Reporting bias assessment	14	Describe any methods used to assess risk of bias due to missing results in a synthesis (arising from reporting biases).	Line 110-112
Certainty assessment	15	Describe any methods used to assess certainty (or confidence) in the body of evidence for an outcome.	Line 112-113
**RESULTS**			
Study selection	16a	Describe the results of the search and selection process, from the number of records identified in the search to the number of studies included in the review, ideally using a flow diagram.	Figure1
	16b	Cite studies that might appear to meet the inclusion criteria, but which were excluded, and explain why they were excluded.	Line27-134
Study characteristics	17	Cite each included study and present its characteristics.	Table1
Risk of bias in studies	18	Present assessments of risk of bias for each included study.	Supplementary Table S3
Results of individual studies	19	For all outcomes, present, for each study: (a) summary statistics for each group (where appropriate) and (b) an effect estimate and its precision (e.g. confidence/credible interval), ideally using structured tables or plots.	Figure 2,3
Results of syntheses	20a	For each synthesis, briefly summarise the characteristics and risk of bias among contributing studies.	Supplemnt Table S3

Section and Topic	Item #	Checklist item	Location where item is reported
	20b	Present results of all statistical syntheses conducted. If meta-analysis was done, present for each the summary estimate and its precision (e.g. confidence/credible interval) and measures of statistical heterogeneity. If comparing groups, describe the direction of the effect.	Line 148-182
	20c	Present results of all investigations of possible causes of heterogeneity among study results.	Table 2
	20d	Present results of all sensitivity analyses conducted to assess the robustness of the synthesized results.	Supplement Table S5
Reporting biases	21	Present assessments of risk of bias due to missing results (arising from reporting biases) for each synthesis assessed.	Line 173-178
Certainty of evidence	22	Present assessments of certainty (or confidence) in the body of evidence for each outcome assessed.	Line 183-193
**DISCUSSION**			
Discussion	23a	Provide a general interpretation of the results in the context of other evidence.	Line 195-249
	23b	Discuss any limitations of the evidence included in the review.	Line 256-259
	23c	Discuss any limitations of the review processes used.	Line 259-262
	23d	Discuss implications of the results for practice, policy, and future research.	Line 263-268
**OTHER INFORMATION**			
Registration and protocol	24a	Provide registration information for the review, including register name and registration number, or state that the review was not registered.	N/A
	24b	Indicate where the review protocol can be accessed, or state that a protocol was not prepared.	N/A

Section and Topic	Item #	Checklist item	Location where item is reported
	24c	Describe and explain any amendments to information provided at registration or in the protocol.	N/A
Support	25	Describe sources of financial or non-financial support for the review, and the role of the funders or sponsors in the review.	N/A
Competing interests	26	Declare any competing interests of review authors.	Line 275-276
Availability of data, code and other materials	27	Report which of the following are publicly available and where they can be found: template data collection forms; data extracted from included studies; data used for all analyses; analytic code; any other materials used in the review.	N/A

Table S2. Strategies for the database search.

Search number	Query
4	#8 and #9 and #10
3	((National Institutes of Health Stroke Scale) OR (severity)) OR (NIHSS)
2	((((((((((((((("Bilirubin"[Mesh]) OR (Bilirubin IX alpha)) OR (Bilirubin, (4E)-Isomer)) OR (Bilirubin, (4E,15E)-Isomer)) OR (Hematoidin)) OR (Bilirubin, Disodium Salt)) OR (Disodium Salt Bilirubin)) OR (Bilirubin, Monosodium Salt)) OR (Monosodium Salt Bilirubin)) OR (delta-Bilirubin)) OR (Bilirubin, (15E)-Isomer)) OR (delta Bilirubin)) OR (Bilirubin, (15E)-Isomer)) OR (Bilirubin, Calcium Salt)) OR (Calcium Salt Bilirubin)) OR (Salt Bilirubin, Calcium)) OR (Calcium Bilirubinate)) OR (Bilirubinate, Calcium)
1	((((((((((((((((((((("Ischemic Stroke"[MeSH Terms]) OR (Ischemic Strokes)) OR (Stroke, Ischemic)) OR (Ischaemic Stroke)) OR (Ischaemic Strokes)) OR (Stroke, Ischaemic)) OR (Cryptogenic Ischemic Stroke)) OR (Cryptogenic Ischemic Strokes)) OR (Ischemic Stroke, Cryptogenic)) OR (Stroke, Cryptogenic Ischemic)) OR (Cryptogenic Stroke)) OR (Cryptogenic Strokes)) OR (Stroke, Cryptogenic)) OR (Cryptogenic Embolism Stroke)) OR (Cryptogenic Embolism Strokes)) OR (Embolism Stroke, Cryptogenic)) OR (Stroke, Cryptogenic Embolism)) OR (Wake-up Stroke)) OR (Stroke, Wake-up)) OR (Wake up Stroke)) OR (Wake-up Strokes)) OR (Acute Ischemic Stroke)) OR (Acute Ischemic Strokes)) OR (Ischemic Stroke, Acute)) OR (Stroke, Acute Ischemic)

Table S3. Quality evaluation of included studies.

	Selection				Comparability	Outcome			Final score
**Cohort studies**									
Authors	Representativeness of the exposed cohort	Selection of the non-exposed cohort	Ascertainment of exposure	Demonstration that outcome of interest was not present at start of study	Comparability of cohorts on the basis of the design or analysis	Assessment of outcome	Was follow-up long enough for outcomes to occur	Adequacy of follow up of cohorts	
Li, Z.	1	1	1	1	2	1	1	1	9
Luo, Y.	1	1	1	1	2	1	1	1	9
Pineda, S	1	1	1	1	2	1	1	1	9
**Case-control study**									
Authors	Is the case definition adequate?	Representativeness of the cases	Selection of Controls	Definition of Controls	Comparability of cases and controls on the basis of the design or analysis	Ascertainment of exposure	Same method of ascertainment for cases and controls	Non-Response rate	Final score
Chen Guodong	1	1	1	0	2	1	0	1	7

Yan Wang	1	1	1	1	2	1	1	1	9
Ye Shan	1	1	1	1	2	1	1	1	9

Cross-sectional study

Authors	Representativeness of the cases	Non-respondents	Adequate definition of exposure	Ascertainment of exposure	Comparability on the basis of the design or analysis	The study used a precise definition of outcome and valid and reliable method (individually for each relevant outcome)	Assessment of outcome	Statistical test	Final score
Xu, T	1	1	1	1	2	Unscored	1	1	8

Table S4 . Characteristics of included studies.

**Study ID**	Year	Area	Study design	Source of population	Total number	Outcome	Bilirubin collection time	Bilirubin types	Bilirubin (μmol/L)	Distribution of bilirubin (μmol/L)	Included	Excluded	Adjustment
Pineda, S	2008	USA	Cohort	Hospital-based	743	Stroke severity	After stroke: 24 hours of admission	DBIL	N/A	quartiles	admission for acute ischemic stroke; bilirubin levels obtained on admission; and no established history of hepatic disease	N/A	age, sex, history of atrial fibrillation, history of hypertension, hyperlipidemia, diabetes, smoking status, admission glucose, premorbid antithrombotic use, premorbid statin use, and premorbid functional status.
Luo, Y.	2012	China	Cohort	Hospital-based	531	Stroke severity	After stroke: on hospital admission	TBIL DBIL	TBIL: 18.54±0.40 μmol/L DBIL: 4.70±0.10 μmol/L	quartiles	At admission, plain CT scan of the head was done to rule out haemorrhage and MRI was done to	Without CT of the head; Without MRI of the head; Admitted more than 7 days after the onset of symptoms; Pre-stroke impairment; Coexistence with infections diseases; Coexistence with	blood glucose (BG), uric acid (UA), Triglyceride (TG), cholesterol(TC), High density lipoprotein cholesterol (HDL-C), Low

											identify the new infarction	inflammatories diseases; Coexistence with hepatic diseases; Coexistence with renal diseases; Coexistence with tumor;	density lipoprotein cholesterol (LDL-C)
Xu, T.	2013	China	Cross-sectioal	Hospital-based	2361	Stroke severity	After stroke: 24 hours of admission	TBIL DBIL	TBIL: 17.57±9.36 μmol/L DBIL: 3.53±6.56 μmol/L	quartiles	confirmed by a computed tomography (CT) scan or magnetic resonance imaging (MRI)	missing data of bilirubin or other covariates.	age, sex, alcohol consumption, ciga- rette smoking, blood levels of glucose and lipids, admission SbP and DbP, blood urea nitrogen, serum creatinine, sodium, hematocrit, history of stroke, hypertension, diabetes, coronary heart disease, rheumatic heart disease, and atrial fibrillation, family history of stroke, hypertension and dia- betes

Ye Shan	2016	China	Case-control	Hospital-based	290	Stroke severity	After stroke: 2 days of admission	TBIL	TBIL: 14.968± 6.613 μmol/L	N/A	Met the diagnostic criteria of the Chinese guidelines for the diagnosis and treatment of acute ischemic stroke 2010, had definite symptoms and signs of focal neurological deficits, and were confirmed by MRI; (2) Age > 18 years; (3) Visits within 72 h of onset	Transient ischemic attack (TIA), intracerebral hemorrhage, brain tumor, or brain trauma; Concomitant diseases including liver, biliary, pancreatic, renal diseases, neoplasms as well as drug-induced liver injury may affect bilirubin metabolism	N/A
Chen Guodong	2016	China	Case-control	Hospital-based	108	Stroke severity, Short term prognos	After stroke: 24 hours of admission	TBIL DBIL IBIL	TBIL: 18.52±7.61 μmol/L DBIL:	quartiles	They met the diagnostic criteria according to the 2010	Liver function damage due to various causes, hemolytic jaundice, obstructive jaundice, hemorrhagic cerebral	Type 2 diabetes mellitus, fasting plasma glucose, systolic blood pressure,

						is			3.82±1.32 μmol/L IBIL: 14.43±5.23 μmol/L		guidelines for the diagnosis and treatment of acute ischemic stroke in China and were confirmed by cranial CT and / or MRI; Age ≥ 50 years;First occurrence of stroke, and a visit within 3 d of onset; Well established and detailed clinical data are available	infarction and other hemorrhagic diseases, infectious diseases, severe kidney disease, autoimmune diseases, malignancies, etc; Use of sulfonamides, bile acid lowering, choleretic drugs, salicylic acid and other drugs that affect bilirubin metabolism; With severe anemia. For the control group, contemporaneous health check ups with similar gender, age, and vascular risk factors were selected, and liver, biliary, renal, tumor, and hematologic history were excluded	low-density lipoprotein cholesterol level
Yan Wang	2018	China	Case-control	Hospital-based	73	Stroke severity	After stroke: on hospital admission	TBIL DBIL IBIL	TBIL: 25.62±12.33 μmol/L DBIL:	N/A	(a) patients met the diagnostic criteria for a cerebral	patients with severe infection, cerebral tumors, hematological disorders, and autoimmune disease	N/A

8.72±5.15 μmol/L IBIL: 16.84±7.96 μmol/L	infarction established by the Chinese Medical Association Neuropathy Academy and TOAST classification system; were admitted to the hospital within 48 hours from the onset of stroke; and had not received a treatment of thrombolytic therapy	confirmed by clinical study, CT, or biochemical tests that could result in a nonatherosclerotic cerebrovascular disease; patients with auricular fibrillation, atrial flutter, heart-valve disease, and myocardial infarctions within four weeks of the stroke which could lead to cardiogenic cerebral infarctions; patients with severe dysfunction of the heart (arrhythmia, cardiac failure, myocardial infarction, or myocarditis), liver (hepatocyte degeneration and necrosis, increased glutamic-pyruvic transaminase, and bilirubin), and kidneys (blood urea nitrogen > 6.8 mmol/L, serum creatinine > 159.1

												μmol/L, blood urea > 7.0 mmol/L); patients with central nervous system infections, which had inflammatory response, myelinoclasis or meningitis; patients with a history of perinatal strokes; patients being treated with thrombolytic therapy; patients with lesions detected by imaging examinations with an absenceof clinical symptoms; patients who could not be examined with a head MRI due to a cardiac pacemaker, heart stent, middle ear implant, or metallic intraocular foreign body were all excluded from this study.	
Li, Z.	2020	China	Cohort	Hospital-based	610	Stroke severity	After stroke: on hospital admission	TBIL DBIL	TBIL: 18.313± 9.432 μmol/L	N/A	At admission, plain CT scan of the head was done to	N/A	high density lipoprotein cholesterol, low density lipoprotein

	DBIL: 4.706±2 .541 μmol/L	rule out haemorrhage and MRI was done to identify the new infarction		cholesterol, Total cholesterol, triglyceride

TBIL=total bilirubin；DBIL= Direct bilirubin；IBIL= Indirect bilirubin

Table S5. Sensitivity analysis on the pooled ORs of ischemic stroke severity.

Study omitted	OR	95% CI	
		Lower CI Limit	Upper CI Limit
Total bilirubin			
Li, Z. (2020)	1.13	1.09	1.18
Li, Z. (2020)	1.16	1.10	1.23
Luo, Y. (2012)	1.12	1.08	1.16
Xu, T. (2013)	1.12	1.08	1.15
Direct bilirubin			
Li, Z. (2020)	1.10	1.06	1.14
Li, Z. (2020)	1.09	1.05	1.14
Luo, Y. (2012)	1.10	1.06	1.14
Pineda, S (2008)	1.71	1.58	1.85
Xu, T. (2013)	1.09	1.05	1.13

Table S6. Differences in bilirubin levels between moderate and severe stroke patients on day 1 and 14 after admission.

Study ID	Day 1, Bilirubin (μmol/L)	Bilirubin (μmol/L)	WMD 95%CI
Total bilirubin			
Chen Guodong	20.85±6.82	16.62±6.58	4.23(1.49-6.97)
Yan Wang	36.1±2.8	17.1±3.5	19.00(17.63-20.37)
**Overall**			**11.66(-2.81-26.14)**
Direct bilirubin			
Chen Guodong	3.84±1.32	3.35±1.25	0.49(-0.01-0.99)
Yan Wang	12.8±3.5	7.1±2.2	5.70(4.39-7.02)
**Overall**			**3.06(-2.05-8.16)**
Indirect bilirubin			
Chen Guodong	16.28±4.52	12.56±4.96	3.72(1.78-5.66)
Yan Wang	23.3±5.2	8.7±3.1	13.30(11.31-15.29)
**Overall**			**8.51(-0.88-17.90)**
